# A National Database Retrospective Review of Short-Term Postoperative Mortality in the Geriatric Population: A Comparison Between Emergency Spine Fractures and Hip Fractures

**DOI:** 10.7759/cureus.55038

**Published:** 2024-02-27

**Authors:** Bongseok Jung, Alex Ngan, Sarah Trent, Austen Katz, Sohrab Virk, David Essig

**Affiliations:** 1 Orthopedic Spine Surgery, Northwell Health, Manhasset, USA; 2 Orthopedics, Northwell Health, Manhasset, USA; 3 Orthopedic Surgery, Northwell Health, Hempstead, USA

**Keywords:** national surgical quality improvement program (nsqip), emergency, mortality, hip fracture, spine fracture

## Abstract

Background: Mortality rates following emergency spine fracture surgery are high, especially in the elderly. However, how the postoperative mortality rate following spine fractures compares to other geriatric fractures such as hip fractures remains unclear. Therefore, this retrospective cohort study aimed to compare 30-day mortality rates and risk factors between emergency spine fracture versus hip fracture surgery in the elderly.

Methods: The National Surgical Quality Improvement Program (NSQIP) database was queried between 2011 and 2021 for emergency spine fractures and hip fractures in the elderly. Univariate analyses evaluated demographic data, perioperative factors, comorbidities, and 30-day mortality rates as the primary outcomes. A multivariable regression model was then constructed to control for significant baseline and demographic differences and evaluate independent predictors of mortality.

Results: A total of 18,287 emergency hip fractures and 192 emergency spine fractures were included in our study. Univariate analysis demonstrated significant differences in female sex, body mass index (BMI), operation time, length of hospital stays, disseminated cancer, and functional dependence between spine and hip fractures. Thirty-day mortality rates were significantly higher in spine versus hip fractures (9.4% vs. 5%). Multivariate regression analysis demonstrated emergent spine fracture surgery, disseminated cancer, functional dependence, and length of stay as independent predictors of mortality in our cohort. Female sex, BMI, and operation time were protective factors for mortality in our cohort.

Conclusions: Emergency spine fractures in the elderly represent an independent predictor for 30-day postoperative mortality compared to emergency hip fractures. Disseminated cancer, functional dependence, and length of stay were independent predictors of mortality while female sex, BMI, and operation time were protective factors. These data demonstrate the severity of injury and high rates of mortality that clinicians can use to counsel patients and their families.

## Introduction

Spine fractures and hip fractures are common geriatric events that are significant public health concerns for the elderly population. Within the United States, hip fractures were found to have an annual mean incidence rate per 100,000 of 957.3 in women and 414.4 in men [1]. Spine fractures are highly prevalent in the elderly, suggested to be between 11% and 18% in patients aged 70 and older [2]. There are significant rates of mortality associated with hip and spine fractures; the one-year mortality rate among elderly patients following hip fractures is estimated to be between 15% and 35% [3]. Similar outcomes have been observed in elderly spine fracture patients, with an estimated one-year mortality rate of approximately 10%-36% [4].

There is a significant healthcare burden related to hip and spine fractures. In 2019, hip fractures were identified as costing the US healthcare system $5.96 billion annually, with projections indicating an expected increase to $18.2 billion by 2025 [5]. Individual patient quality of life may be significantly affected by the costs associated with acute pain and limitations to mobility. An estimated 40% to 70% of patients can restore mobility to their pre-fracture levels and approximately 59% report pain at one-year follow-up [6]. Comparatively, spinal fractures were found to have an aggregate cost of surgery of approximately one billion dollars in the United States in 2014 [7]. Symptoms of spinal fractures are comparatively moderate in severity as they vary between inconspicuous and debilitating symptom progression.

It has been suggested that the number of hip fractures and spine fractures, both domestically and globally, will continue to increase, due to a growing geriatric population [6,8,9]. Despite the prevalence of these events, there are limited studies directly comparing short-term outcomes between hip and spine fractures. There are currently only two studies directly comparing mortality rates between hip and spinal fracture patients, one of which evaluated patients with end-stage kidney disease and the other comparing one-year mortality and morbidity rates [10,11].

Our study aimed to compare 30-day mortality rates and independent risk factors among elderly patients with emergent spine and hip fractures. We utilized data from the National Surgical Quality Improvement Program database to query 30-day outcomes and evaluate comorbidities associated with mortality rates while comparing the two types of fractures. Additionally, ours is the first study to identify independent risk factors for mortality associated with both orthopedic fractures.

## Materials and methods

This study was a retrospective cohort analysis using the American College of Surgeons National Surgical Quality Improvement Program (ACS-NSQIP), a free national public database often used for retrospective reviews in orthopedic surgery. As the database only included de-identified patient information that is publically available, it carries minimal risks for individual subjects that were involved. Therefore, the present study was exempt from review by the local Institutional Review Board (IRB) per the ethical principles of anonymity, minimal risks, and confidentiality.

Cases during the operation years between 2011 and 2021 were queried using Current Procedural Terminology (CPT). Inclusion criteria included CPT codes for cervical, lumbar, and thoracic spine fractures (22325, 22326, and 22327, respectively) and hip fractures (27326, 27244, and 27245). Other inclusion criteria included all elderly patients (≥65 years) and emergent cases as defined by the NSQIP database. Exclusion criteria included any known missing, unknown, or unclear demographic variables. Additionally, all patients aged <65 years, or any elective cases, urgent cases, and non-emergent cases were excluded from the study.

Patient demographics, perioperative factors, comorbidities, and 30-day mortality rates were collected from the database and analyzed. Demographic variables that were compared included age (years), female sex, non-white race, Hispanic ethnicity, and body mass index (BMI) (kg/m^2^). The perioperative factors analyzed included operation time (minutes), outpatient surgery, length of stay (days), and non-home discharge. Comorbidities included current smoking, chronic obstructive pulmonary disease (COPD), congestive heart failure (CHF) 30 days before surgery, dialysis, disseminated cancer, functional dependence, chronic steroid use, and bleeding disorder. Finally, 30-day mortality rates were compared between emergent hip fractures and spine fractures.

Statistical analysis was carried out using IBM SPSS Statistics for Windows, Version 28.0 (IBM Corp., Armonk, NY). The baseline characteristics of our cohorts were first characterized using descriptive statistics for continuous variables and frequencies for categorical variables. The variables were compared using the chi-squared test for categorical variables and independent two-sample t-tests for continuous variables, as deemed appropriate. Significance was set at a *P*-value less than 0.05.

To address notable differences in baseline characteristics, a multivariable regression model for mortality was developed using a backward stepwise approach, with entry at a *P*-value of 0.05 and removal at a *P*-value of 0.10. All variables that remained in the model were considered significant variables affecting mortality. Results from this multivariable regression were reported as odds ratio with 95% confidence intervals (OR ± 95% CI), with a *P*-value less than 0.05 as significant.

## Results

A total of 18,479 patients were included in our study, with 18,287 (98.96%) hip fractures and 192 (1.04%) spine fractures (Table 1). Among the baseline characteristics, female sex, BMI, operation time, length of hospital stay, disseminated cancer, and mortality were significantly different between patients with emergent hip fractures versus spine fractures. In patients with emergent spine fractures, there were fewer female sex, longer operation times, longer hospital stays, higher rates of disseminated cancer, and higher mortality rates compared to hip fractures. Therefore, these significant baseline characteristics were controlled for in the multivariable regression analysis. 

Limitations of the NSQIP database should be acknowledged. These include the inability to stratify certain comorbidities analyzed in our study, such as the length of prior smoking history, the severity of COPD and heart failure, and the chronicity of past steroid use. Additionally, this database may contain biases as it is limited to American patients and the hospitals that have volunteered to be included in the database. 

**Table 1:** Baseline differences between emergent hip fracture and spine fracture surgery in the elderly. BMI, body mass index; COPD, chronic obstructive pulmonary disease; CHF, congestive heart failure

	Hip fracture	Spine fracture	*P*-value
Total cases (*n* = 18,479)	18,287 (98.96%)	192 (1.04%)	
Demographic variables			
Age (years)	80.0 ± 6.7	74.8 ± 6.7	0.501
Female sex	12,752 (69.7%)	81 (42.2%)	<0.001
Non-white race	1,600 (8.7%)	22 (11.5%)	0.187
Hispanic ethnicity	994 (5.4%)	13 (6.8%)	0.417
BMI (kg/m^2^)	24.1 ± 7.6	28.0 ± 8.2	<0.001
Perioperative factors			
Operation time (minutes)	67.9 ± 40.8	193.8 ± 98.5	<0.001
Outpatient surgery	101 (0.6%)	1 (0.5%)	0.953
Length of stay (days)	5.5 ± 4.0	10.9 ± 7.4	<0.001
Non-home discharge	14,807 (81%)	149 (77.6%)	0.238
Comorbidities			
Current smoker	2,048 (11.2%)	23 (12.0%)	0.733
COPD	2,349 (12.8%)	20 (10.4%)	0.317
CHF 30 days before surgery	755 (4.1%)	7 (3.6%)	0.738
Dialysis	432 (2.4%)	5 (2.6%)	0.826
Disseminated cancer	453 (2.5%)	15 (7.8%)	<0.001
Functional dependence	3,569 (19.5%)	22 (11.5%)	0.005
Chronic steroid use	995 (5.4%)	16 (8.3%)	0.08
Bleeding disorder	3,317 (18.1%)	25 (13.0%)	0.067
30-day outcome			
Mortality	915 (5.0%)	18 (9.4%)	0.006

Finally, binary logistic multivariate regression analysis to control for significant baseline characteristics was performed to evaluate independent predictors of mortality following hip versus spine fractures (Table 2). Our model demonstrated that female sex, BMI, and operation time were protective factors of mortality in our cohort. Length of hospital stay post-operation, functional dependence status, disseminated cancer, and emergent spine fracture surgery were significant independent predictors of mortality in our cohort.

**Table 2:** Odds ratio with 95% CI of independent risk factors for mortality between emergent hip versus emergent spine fractures in the elderly. BMI, body mass index; CI, confidence interval

	Odds ratio	95% CI
Female sex	0.535	0.466-0.613
BMI (kg/m^2^)	0.981	0.973-0.989
Operation time (minutes)	0.998	0.996-1.000
Length of stay (days)	1.047	1.036-1.059
Functional dependence	2.893	2.518-3.325
Disseminated cancer	3.97	3.075-5.126
Spine fracture surgery	1.828	1.046-3.193

## Discussion

This study aimed to compare 30-day mortality rates and identify risk factors for mortality following emergent spine fracture versus hip fracture surgery in the elderly. Results revealed that emergent spine fractures were associated with a higher rate of 30-day mortality compared to emergent hip fractures in the elderly. Furthermore, spine fracture surgery, along with disseminated cancer, functional dependence, and length of stay in the hospital were independent risk factors for 30-day mortality when compared to emergent hip fracture surgery. Female sex, BMI, and operation time were found to be protective factors of mortality in our cohort. 

To the authors’ knowledge, this is the first paper to assess and compare the risk factors associated with mortality in spinal and hip fractures. Furthermore, ours is one of few studies that aimed to directly compare emergency spine and hip fractures, especially risk factors and 30-day mortality rates in elderly patients. Results demonstrate a significantly higher mortality rate in emergent spine fractures when compared to emergent hip fractures (9.4% and 5.0%, respectively). Spine fracture surgery was also a significant predictor of 30-day postoperative mortality compared to hip fractures. These results fall within the range of mortality rates previously established in the literature that evaluated these events independently [12,13]. However, our results differ from the current literature which compared hip and spine fracture outcomes. One systematic review and meta-analysis by Shimamura et al. compared hip and spinal fractures in patients with end-stage kidney disease. They found a one-year mortality rate of 27% and 10% for hip and spine fractures, respectively [11]. Another study by Rizkallah et al. reported a one-year mortality rate of 32% and 11% for hip and spine fractures, respectively [10]. This may be attributed to our evaluation of the mortality rate at 30 days compared to one year, which may potentially indicate that patients with emergent spinal fractures experience relatively higher mortality rates in the immediate postoperative period following surgery compared to hip fractures.

There is extensive literature considering the various risk factors associated individually with post-hip fractures and individually with post-spinal fracture mortality in elderly patients. Potential risk factors have been found to range from demographic to environmental factors that are significantly correlated with mortality. A retrospective review by Barceló et al. found that age, male sex, comorbidity, delirium, and medical complications were risk factors for mortality following hip fractures [14]. Furthermore, Ek et al. found that length of hospital stay was associated with increased mortality [15]. Spinal fractures have similar risk factors for mortality. Gutiérrez-González et al. reported that age, male sex, oncological history, and comorbidity were independent risk factors for post-spinal fracture mortality [16]. Soon et al. found that mobility status and the presence of old compression fractures were also factors that influence spinal fracture mortality [4]. Our study was the first to compare risk factors of mortality in spine fractures compared to hip fractures. Results suggest that disseminated cancer, functional dependence, and length of hospital stay are independent predictors of mortality which agrees with the aforementioned literature.

These risk factors for mortality align with the previously reported causes of death for both hip and spinal fractures. A retrospective review of patients by Barceló et al. found that pneumonia, circulatory system diseases, and dementia were the leading causes of death within the first two years [14]. Panula et al. identified circulatory disease, dementia, and Alzheimer’s disease as the leading causes of death following hip fracture with a mean follow-up of 3.7 years [17]. On the other hand, Soon et al. examined mortality for geriatric spinal fractures and found pneumonia and ischemic heart disease to be the most prevalent causes of death at 3, 6, and 12 months [4]. Our results suggest that within the first 30 days postoperatively, geriatric patients may be more susceptible to mortality following spinal fracture surgery compared to hip fractures. Thus, emergency spinal fracture surgeries may have a comparatively higher risk for acute intraoperative and postoperative complications that predispose patients to mortality. Future studies should therefore aim to compare peri and postoperative factors such as acute circulatory and pulmonary complications of spine fractures when compared to emergency hip fracture surgery in the geriatric population.

In the context of patient care and decision-making, these findings demonstrate the severity of injury and high rates of mortality associated with emergent spine fractures that clinicians can use to counsel patients and their families. The data suggests that elderly patients may have a greater risk of mortality after undergoing surgery for emergent spine fractures versus if they were to have emergent hip fractures in the short term postoperatively. This can therefore be used to help guide clinical judgments to provide the best form of care that is inclusive of a patient's ultimate goals of care. It may also help inform the risk and benefit conversations both before and after the surgery for a patient and their family. 

The limitations of the study should also be discussed. The population of spine fracture patients in this study was statistically appropriate but smaller compared to hip fracture patients. Our patient population allowed for robust statistical analyses and accounted for discrepancies in sample sizes. However, utilization of databases beyond ACS-NSQIP would allow for a larger sample size of spinal fracture patients and comparison of additional patient data. The strengths of the study should also be recognized. Our study included a large sample size (*n* = 18,479) from a national database and comprehensively analyzed several risk factors that may be associated with spine fractures. Our study was also the first to evaluate independent risk factors associated with a comparison of spine versus hip fractures, which provided insight into the high risk of mortality that may be associated with emergent spine fractures in elderly patients.

Future studies should, therefore, include additional factors that have been shown to correlate with mortality rate such as quality of life before surgery. It should also utilize databases beyond ACS-NSQIP such as the PearlDriver database to allow for more discrete comparisons of comorbidities such as degree of smoking history and medication use in the past. Other databases may also allow for more long-term comparisons such as six-month and one-year mortality rates as opposed to only short-term 30-day mortality rates. This would help clarify whether there is indeed a discrepancy in mortality rates following emergent spine and hip fractures in the short term versus the long term. Finally, a comparison of hip fractures versus other types of fractures outside of hip fractures may yield a more comprehensive picture of how the data fits in with other types of injuries in the geriatric population.

## Conclusions

Short-term mortality rates in emergent spinal fractures were found to be significantly greater than those of emergent hip fractures in the geriatric population. Overall, the data demonstrate the severity of injury and high rates of mortality associated with emergent spinal fractures in the geriatric population that clinicians can use to counsel patients and their families. Future larger and more comprehensive studies should aim to assess the discrepancies between emergent spinal fractures versus other fractures in the elderly population.
